# Editorial: Biological Phase Separation

**DOI:** 10.3389/fgene.2022.895689

**Published:** 2022-04-13

**Authors:** Eiichiro Mori, Tony Z. Jia

**Affiliations:** ^1^ Department of Future Basic Medicine, Nara Medical University, Nara, Japan; ^2^ Earth-Life Science Institute, Tokyo Institute of Technology, Tokyo, Japan; ^3^ Blue Marble Space Institute of Science, Seattle, WA, United States

**Keywords:** biological phase separation, liquid-liquid phase separation (LLPS), membrane-less organelle, stress response, self-interaction, multivalent interaction

In cells, the nucleus and mitochondria are surrounded by lipid membranes, but there are also organelles not surrounded by membranes, such as nucleoli and RNA granules. The biophysical mechanism to explain how these membrane-less organelles are formed is called liquid-liquid phase separation (LLPS), applied from classical physical chemistry. As LLPS in classical physical chemistry and phase separation in cells are considered to be distinct in some ways, “biological phase separation” might be more appropriate to describe this phenomenon.

LLPS defines distinct compartments to efficiently organize cellular processes by concentrating on certain factors in their proper location without interfering with one another in the complex and the heterogeneous environment within a cell. Accumulating evidence demonstrates that these LLPS-mediated molecular compartments are required for signal transduction, regulation of gene expression, stress response, and many other aspects of cellular physiology. Saito and Kimura reviewed how cells utilize LLPS to deal with oxidative stress, especially related to cell survival or pathogenesis. Zaepfel and Rothstein demonstrated that the proteins G3BP1/2, which are common stress granule components, are not actually required for the formation of stress granules specifically during osmotic stress induced by sorbitol and related polyols. These studies suggest that biological phase separation may be a flexible and dynamic process, and perhaps there is more than one pathway phase separation of the same cellular membrane-less organelles.

Aberrant regulation of LLPS is often found in neurological and developmental diseases and human genetics offers more information about the physiological roles of LLPS. However, little is known about the mechanisms of formation and regulation of LLPS. In contrast to conventional biomolecular interactions, weak, transient and multivalent interactions drive LLPS formation. Shinkai et al. reviewed the phenylalanine-glycine (FG) repeat proteins that are known to phase separate in the cell. They discuss that these repeat proteins are not only member of the nuclear pore complex, nucleoporins containing a FG repeat domain, but also act as an activator of barrier formation and homotypic cell-cell interactions. Su et al. described how RNA contributes to the biogenesis, dissolution, and other properties of biomolecular condensates as a multivalence-providing scaffold for proteins/RNA to undergo phase separation, especially for N6-methyladenosine (m6A)-containing RNA. m6A is the most widely distributed dynamic post-transcriptional modification and its presence in RNA changes the charge, conformation, and RNA-binding protein anchoring of the modified RNA. Mimura et al. showed the role of the constituent DNA components, i.e., the phosphate groups, deoxyribose sugars, and nucleobases, in LLPS with a polycationic peptide, linker histone H1, a known key regulator of chromatin condensation. These studies show that the chemistry of the LLPS components and the resulting chemical interactions can have significant impacts on LLPS structure, assembly, and function.

While many recent studies (including those in this special issue) have discovered new features of LLPS, to better understand the unconventional behavior of LLPS-forming macromolecules, much more technical advancement of biophysical analytical approaches is desired. Nakasone and Terazima reviewed a novel time-resolved diffusion technique which has a potential to detect molecular events associated with LLPS. Nomoto et al. investigated the parameters of the solubilities of aromatic amino acids in amino acid solvents (PSAS), and proposed a new index based on this parameter that can distinguish amino acids that contribute to droplet and aggregate formation, providing a deeper understanding of LLPS and aggregation of proteins. Masukawa et al. demonstrated that single, uniform DNA hydrogel particles can form inside aqueous/aqueous two-phase systems (ATPSs) assembled in a microwell array. These studies utilize or highlight novel approaches to studying and understanding biological phase separation, and we eagerly wait for other novel techniques that will be developed in the future. In particular, the relevance of LLPS is not restricted only to eukaryotic cells, but also to bacteria and to the origins of life, as membrane-less droplets formed by LLPS have been proposed to have functioned as primitive minimal compartments (i.e., protocells) on early Earth. Thus, as LLPS research is an interdisciplinary endeavor, incorporation and application of techniques from related fields are needed to understand the full picture of LLPS structure, assembly, and function.

